# The influence of multi-morbidity and self-reported socio-economic standing on the prevalence of depression in an elderly Hong Kong population

**DOI:** 10.1186/1471-2458-8-119

**Published:** 2008-04-14

**Authors:** Samuel YS Wong, Stewart W Mercer, Jean Woo, Jason Leung

**Affiliations:** 1 Department of Community & Family Medicine, The Chinese University of Hong Kong, 4/F, School of Public Health, Prince of Wales Hospital, Shatin, N.T., Hong Kong; 2 Department of Medicine & Therapeutics, The Chinese University of Hong Kong, 9/F, Clinical Sciences Building, Prince of Wales Hospital, Shatin, N.T., Hong Kong; 3 Jockey Club Center for Osteoporosis Care and Control, The Chinese University of Hong Kong, 3/F, School of Public Health, Prince of Wales Hospital, Shatin, N.T., Hong Kong; 4 General Practice and Primary Care, Division of Community-Based Sciences, Faculty of Medicine, University of Glasgow, UK

## Abstract

**Background:**

There has been an increasing prevalence of both depression and chronic medical conditions globally but the relationship between depression and multi-morbidity is not well understood. The aim of the present study was to investigate the relationship between depression, multi-morbidity (number of chronic medical conditions, and measures of socioeconomic standing (SES) in an elderly Hong Kong population.

**Methods:**

Cross sectional study. Information on clinically relevant depressive symptoms, measured by the Geriatric Depression Scale (GDS), and demographic and chronic medical conditions were collected using standardized questionnaires. Information collected on SES included educational status (ES), maximum ever income (MEI), and self-perceived social standing in local community (SES-COM) and in Hong Kong generally (SES-HK). Analysis was conducted using multiple logistic regression

**Results:**

Depression rates were similar in men and women (GDS caseness 8.1% vs 8.4%). Multi-morbidity of chronic medical conditions was common (40% of men and 46% of women had three or more). In the overall sample, the prevalence of depression was associated with the number of chronic medical conditions (OR 1.27; CI: 1.16–1.39). In addition, SES-HK and SES-COM were significant independent variables.

**Conclusion:**

In this elderly Hong Kong population, depression prevalence rose markedly with number of chronic medical conditions and SES-HK and SES-COM.

## Background

Mental illness is a significant public health problem worldwide. According to the World Health Organization [1], by 2010, depression will be the second most important condition globally in terms of disability (Disability Adjusted Life Years). It will also be the disease with the greatest burden on society in developed countries [1].

Mental health problems, physical conditions and socio-economic status appear to be closely related in many societies [2-4]. For people with low socioeconomic status, association with increased morbidity and mortality rates have been documented [3,4]. Patients with chronic medical conditions are at increased risk of significant psychological distress including depression, resulting in role functioning impairment [5-8], increase in treatment costs [9], decrease in compliance with medical regimens [10] and worsened disease course leading to higher mortality and disability [11,12]. In the area of mental health, patients with low socioeconomic status or those who live in deprived areas often have a higher prevalence and incidence of depression and psychological distress [13-15]. Although these associations between depression and chronic diseases are well recognized [16], the possible synergistic influence of co-morbidity and deprivation on psychological distress is less well documented [17,18].

In Hong Kong, studies conducted in the elderly have demonstrated a significant association between depressive symptoms and a number of different chronic conditions including osteoporosis [19], chronic obstructive pulmonary diseases [20], lower urinary tract symptoms [21] and stroke [22]. The current study was conducted to investigate the relationship between both objective and subjective socio-economic standing, multi-morbidity, and depression in an elderly Chinese population.

## Methods

Data from 3394 men and women aged 65 years and over who were parts of a study on risk factors of osteoporosis were used in the current study. Subjects were recruited by placing notices in community centres for the elderly and housing estates, followed by talks that explained the purpose, procedures and investigations of the study. Subjects were volunteers with the aim to recruit a stratified sample so that approximately 33% were in each of these age groups: 65–69, 70–74, 75+. The study was approved by the Clinical Research Ethics Committee of the Chinese University of Hong Kong, which requires written informed consent to be obtained.

A questionnaire containing information regarding demographics, socioeconomic status, medical history, self perceived social standing and depressive symptoms was administered by a trained interviewer. The presence or absence of disease was based on subjects' report of diagnosis by their doctors on 14 common chronic medical conditions (LTC). Socio-economic status was assessed both subjectively and objectively. For subjects' self perceived socioeconomic status, an instrument developed by the John D and Catherine T. MacArthur Research Network on Socioeconomic Status and Health was used (Adler et al, 2000). Subjects were asked to place a mark on an upright ladder with ten rungs, with the lowest rung being the most undesirable and the highest the most desirable state with respect to their standing in the community (community ladder). At the same time, they were also asked to rate themselves by placing a mark on another ladder, the top rung representing people who have the most money, the most education, and the most respected jobs, and the bottom rung representing people at the other extreme (Hong Kong ladder). Education levels and the highest income ever obtained were used as the measure for objective socioeconomic status. Clinically significant depressive symptoms were measured using the validated Chinese version of Geriatric Depression Scale [23] with a cut off score of 8 indicative of clinically relevant depressive symptoms. In the study conducted by Lee et al. [23], the 8-symptom cut off point yielded 96% sensitivity and 87.5% specificity for a DSM-based clinician diagnosis of depression.

### Statistical Analysis

The means of variables between men and women were compared by two sample t-tests for continuous variables and chi-squared tests for categorical variables. The associations between number of chronic medical conditions, SES and depression were studied using independent t-test for continuous variables and chi-squared tests for categorical variables. To study factors associated with depression, logistic regression analysis was conducted to study the independent effects of age, number of chronic medical conditions, marital status, education levels, maximum incomes and self perceived SES on clinically relevant depressive symptoms. All statistical analyses were performed using the statistical package SAS, version 8.02 (SAS Institute, Inc., Cary, North Carolina).

## Results

The characteristics of the overall sample are shown in Table 1. The age distribution was fairly evenly divided between the three age groups (65–69, 70–74, 75 or above) and was similar for men and women. Depression scores and prevalence rates were similar in men and women with approximately 1 in 12 subjects reaching GDS-caseness. Multi-morbidity was common in both genders but more women reported three or more chronic medical conditions than men (46% versus 40%). Significant differences existed between men and women in terms of marital status, with more women being widowed. Educational level and maximum income were also markedly lower in women than men. Self-perceived SES also differed with gender, with men reporting lower SES-HK and SES-COM than women, despite their higher educational status and maximum income. This gender difference was most marked for SES-COM.

**Table 1:** Characteristics of the sample that include gender, age, number of chronic diseases, socio-economic factors and depression

	**Prevalence/Mean(SD)**			
		
**Variables**	**Total (n = 3394)**	**Male (n = 1901)**	**Female (n = 1493)**	p-value*
Male	1901 (56.0%)			

Female	1493 (44.0%)			

Age				0.1061
65–69	1211 (35.7%)	649 (34.1%)	562 (37.6%)	
70–74	1204 (35.5%)	689 (36.2%)	515 (34.5%)	
75 or above	979 (28.9%)	563 (29.6%)	416 (27.9%)	
Geriatric Depression Scale	2.9 (2.8)	2.9 (2.8)	2.8 (2.7)	0.2322
Depression (GDS score ≥ 8)	280 (8.3%)	154 (8.1%)	126 (8.4%)	0.7221
No. of chronic diseases				<.0001
0	355 (10.5%)	242 (12.7%)	113 (7.6%)	
1	733 (21.6%)	411 (21.6%)	322 (21.6%)	
2	867 (25.6%)	491 (25.8%)	376 (25.2%)	
3 or more	1439 (42.4%)	757 (39.8%)	682 (45.7%)	
Current marital status				<.0001
Married or living in a married-like relationship	2541 (74.9%)	1680 (88.4%)	861 (57.7%)	
Widowed	705 (20.8%)	144 (7.6%)	561 (37.6%)	
Separated	44 (1.3%)	21 (1.1%)	23 (1.5%)	
Divorced	33 (1.0%)	15 (0.8%)	18 (1.2%)	
Single, never married	71 (2.1%)	41 (2.2%)	30 (2.0%)	
Education level				<.0001
No education	494 (14.6%)	82 (4.3%)	412 (27.6%)	
Primary or below	1791 (52.8%)	1040 (54.7%)	751 (50.3%)	
Secondary or above	1109 (32.7%)	779 (41.0%)	330 (22.1%)	
Max income ^a^				<.0001
0–1999	596 (23.3%)	145 (9.1%)	451 (46.6%)	
2000–5999	579 (22.6%)	292 (18.4%)	287 (29.7%)	
6000–11999	688 (26.9%)	541 (34.1%)	147 (15.2%)	
12000 or above	694 (27.1%)	611 (38.5%)	83 (8.6%)	
SES ladder – Hong Kong	4.5 (1.9)	4.5 (1.8)	4.7 (1.9)	0.0016
SES ladder – community	6.8 (2.2)	6.3 (2.2)	7.3 (2.1)	<.0001

*p-value of t test for continuous variables or chi square for categorical variables where appropriate, comparing male with female

^a^Sample size for male was 1589 (missing value due to 36 refused and 276 don't know), sample size for female was 968 (missing value due to 138 housewife, 6 refused and 381 don't know)

The association of the number of chronic medical conditions with depression and SES is shown in Table 2. In this sample, depression prevalence rose sharply with the number of chronic medical conditions. Depression prevalence was significantly associated with all measures of SES in this sample (Table 2).

**Table 2:** Logistic regression of gender, age, number of chronic medical conditions, social factors on depression

**Variable**	**N**	**Freq. (%)/Mean (SD)**		**Crude Odds ratio (95% CI)**	**Adj. Odds ratio (95% CI) of model 1**^**1**^	**Adj. Odds ratio (95% CI) of model 2**^**2**^
					
		**Controls**	**Depressed **			
N	3394	3114 (91.8%)	280 (8.2%)			
Sex						
Male	1901	1747 (91.9%)	154 (8.1%)	1	1	1
Female	1493	1367 (91.6%)	126 (8.4%)	1.05 (0.82, 1.34)	0.69 (0.47, 1.02)	1.27 (0.83, 1.94)
Age						
65–69	1211	1118 (92.3%)	93 (7.7%)	1	1	1
70–74	1204	1111 (92.3%)	93 (7.7%)	1.01 (0.75, 1.36)	0.78 (0.54, 1.13)	0.75 (0.50, 1.12)
75 or above	979	885 (90.4%)	94 (9.6%)	1.28 (0.95, 1.72)	0.94 (0.64, 1.39)	1.03 (0.67, 1.56)
No. of chronic diseases*	3394	2.3 (1.6)	3.1 (1.7)	1.27 (1.19, 1.36)	1.29 (1.19, 1.41)	1.27 (1.16, 1.39)
Current marital status						
Married or living in a married-like relationship	2541	2358 (92.8%)	183 (7.2%)	1	1	1
Single/Divorced/separated/widowed	853	756 (88.6%)	97 (11.4%)	1.65 (1.28, 2.14)	1.72 (1.20, 2.47)	1.43 (0.97, 2.12)
Education level						
No education	494	445 (90.1%)	49 (9.9%)	1	1	1
Primary or below	1791	1626 (90.8%)	165 (9.2%)	0.92 (0.66, 1.29)	0.97 (0.61, 1.53)	0.95 (0.58, 1.58)
Secondary or above	1109	1043 (94.1%)	66 (6.0%)	0.57 (0.39, 0.85)	0.75 (0.44, 1.25)	1.00 (0.57, 1.77)
Max income						
0–1999	596	545 (91.4%)	51 (8.6%)	1	1	1
2000–5999	579	520 (89.8%)	59 (10.2%)	1.21 (0.82, 1.80)	1.18 (0.78, 1.78)	1.07 (0.68, 1.67)
6000–11999	688	632 (91.9%)	56 (8.1%)	0.95 (0.64, 1.41)	0.97 (0.61, 1.53)	1.07 (0.66, 1.74)
12000 or above	694	664 (95.7%)	30 (4.3%)	0.48 (0.30, 0.77)	0.52 (0.30, 0.90)	0.71 (0.40, 1.26)
SES ladder – HK*	3394	4.7 (1.8)	3.3 (1.8)	0.65 (0.60, 0.70)	-	0.75 (0.67, 0.83)
SES ladder – community*	3394	6.9 (2.1)	5.2 (2.6)	0.71 (0.67, 0.76)	-	0.76 (0.70, 0.82)

*odd ratio representing per 1 unit increase

^1^model 1 includes all variables except SES ladders

^2^model 2 includes all variables

These univariate correlations confirmed the significant relationship between depression and the number of chronic medical conditions and the relationship between depression and all measures of SES.

Marital status and maximum income were significantly associated with the prevalence of depression. However, these relationships disappeared when SES-COM and SES-HK were entered into the regression.

## Discussion

### Main findings

In the present study the relationship between multi-morbidity, objective and subjective socioeconomic status, and depressive symptoms were explored. We found significant associations between multi-morbidity and depressive symptoms and between SES and depressive symptoms. Although associations between individual chronic medical conditions and depression are well known [19-21,23,24], fewer studies have explored multi-morbidity and depression. We found that there was approximately an increased odds of having clinically relevant depressive symptoms when the number of chronic medical condition increases. Previous work in this area has suggested that it is the severity of the illness (the effect on the person's life) rather than the number of diseases that links multi-morbidity with depression [16] and further work is required to link number and type of disease with severity and depression in the current sample. Literature on the impact of social factors such as social support is an important risk factor for depression [25,26].

In terms of the importance of self-perceived SES, Cheng et al [27] investigated the association between self perceived SES and health outcome and showed that self perceived SES may be a more relevant measure for predicting health outcomes in the elderly, particularly for mental health status. Previous studies [27,28] have also shown that the subjective experience of financial strain may be more closely related to health than objective measures of SES. Skapinakis et al. [29] demonstrated that subjective financial strain at baseline was independently associated with depression at follow up in a cohort. Others [2,28,30] have found that differences in relative income standing correlates with health outcomes (including disability, self rated health and psychological functioning) and the effects can be more consistent and stronger with subjective measures of SES than objective indices of SES. Several mechanisms can explain the association between self perceived SES and clinically relevant depressive symptoms [29] that included lack of social support, lack of control over one's environment or chronic stress. Unfavorable social comparison with others [31] can also cause negative effects on mental health.

In Chinese elderly, using the same measure for subjective SES as in the present study, Hu et al. [3] found a positive association between the subjective ranking on the social hierarchy and self rated health and physical functioning. Of interest the present study was the finding that men reported lower SES-HK and SES-COM than that of women, despite their higher education and higher maximum income. This may be related to the cultural expectations and social role of women in this Chinese society.

To summarize the context of the present study, there is a dearth of studies in the international literature that specifically explore the relation between SES, multi-morbidity and depression. As far as we are aware, our study is the first to show a significant relationship between self-perceived SES, multi-morbidity and clinically important depressive symptoms in Chinese elderly. Our findings also add new information on the relationship between self perceived SES measured by the SES ladder, multi-morbidity and clinically relevant depressive symptoms in Chinese elderly.

### Implications for policy and practice

Our results have implications for policy and practice. By studying the increase of the number of medical conditions and its relation to the risk of depression, we provide clinicians with information that can help them better identify those patients with multi-morbidity who are most at risk for depression. We also showed that those with both low SES and multi-morbidity are at the highest risk for depression.

As low self-perceived SES was shown to correlate strongly with depressive symptoms, health policy that can reduce inequity may decrease the impact of multi-morbidity on depressive symptoms. As we are reaching a point where both developed and developing countries suffer from a large burden on non-communicable diseases and disability resulting from mental disorders, any measures that can decrease the impact of non-communicable diseases and mental disorders can reduce the health burden on society. Health promotion and prevention programs that aim to increase social network and self perceived community standing and social interventions and health promotion that decrease chronic disease burden and increase financial support may all be relevant to decrease depression in the elderly population.

### Limitations

One of the major limitations in our study is that this is a cross sectional study. As a result, no causal relationship can be established between SES, multi-morbidity and depression. It can be argued that depression can also cause a downward mobility effects on SES, although most studies conducted have not supported such a contention. Moreover, the directionality of the relationship between self perceived SES and depression is not known and some may argue that depression can cause low self esteem which may affect one's perception of social economic standing in both community and society.

Second, our study relied on self report of chronic medical conditions. As a result, under diagnosis of diseases and misclassification of disease status could not be excluded. However, as we did not study the relationship between specific types of disease and depressive symptoms, misclassification of disease should not significantly affect our results.

Third, we only used a validated scale to measure clinically relevant depressive symptoms, as a result, our results are only relevant to depressive symptoms and not depression per se.

## Conclusion

We found that both low self perceived SES, actual SES, and multi-morbidity are independent predictors of clinically relevant depressive symptoms. The effects of low self perceived SES on depression may be stronger than the effects from objective measure of SES. Further research using a prospective design will further clarify the role of co-morbidity and in particular, self perceived SES on clinically relevant depressive symptoms and outcomes.

## Competing interests

The author(s) declare that they have no competing interests.

## Authors' contributions

SW participated in the design of the study and performed the statistical analysis. SM conceived of the study and participated in its design and coordination and helped to draft the manuscript and revision. JW participated in the design of study, monitored BMD measurement and participated in revision of manuscript. JL participated in statistical analysis. All authors read and approved the final manuscript.

## Pre-publication history

The pre-publication history for this paper can be accessed here:


